# The downregulation of NCXs is positively correlated with the prognosis of stage II–IV colon cancer

**DOI:** 10.1186/s12957-021-02284-5

**Published:** 2021-06-14

**Authors:** Zhixiu Xia, Changliang Wang, Hong Zhang

**Affiliations:** 1 Colorectal Tumor Surgery Ward, Department of General Surgery, Shengjing Hospital of China Medical University, No. 36, San Hao Street, Shenyang, Liaoning People’s Republic of China; 2 The People’s Procuratorate of Liaoning Province Judicial Authentication Center, No. 46, Cong San East Road, Shenyang, Liaoning 110032 People’s Republic of China; 3 Collaborative Laboratory of Intelligentized Forensic Science (CLIFS), No. 46, Cong San East Road, Shenyang, Liaoning 110032 People’s Republic of China

**Keywords:** NCX, Colon cancer, Calcium ion, Prognosis

## Abstract

**Purpose:**

Colon cancer (CC) is a very common gastrointestinal tumor that is prone to invasion and metastasis in the late stage. This study aims to observe the expression of Na^+^/Ca^2+^ exchangers (NCXs) and analyze the correlation between NCXs and the prognosis of CC.

**Methods:**

Specimens of 111 stage II–IV CC patients were collected. We used western blotting, qPCR, and immunohistochemical staining to observe the distributions and expression levels of NCX isoforms (NCX1, NCX2, and NCX3) in CC and distal normal tissues. Cox proportional hazards models were used to assess prognostic factors for patients.

**Results:**

The expression of NCXs in most tumor specimens was lower than that in normal tissues. The NCX expression levels in tumor tissues from the primary tumor, local lymph node metastasis sites, and distant liver metastasis sites were increasingly significantly lower than those in normal tissues. The results of the Kaplan-Meier survival curves showed that the downregulation of any NCX isoform was closely related to the worse prognosis of advanced CC.

**Conclusion:**

NCXs can be used as independent prognostic factors for CC. Our research results are expected to provide new targets for the treatment of CC.

## Introduce

Colon cancer (CC), one of the malignant tumors of the digestive tract, is the third most common cancer and the fourth leading cause of cancer-related death worldwide [1]. Patients with early-stage CC have a better prognosis after surgery. However, those with late-stage CC often develop local invasion and distant metastasis, which leads to a poor prognosis [2, 3]. Many studies have confirmed that the excessive opening of some calcium (Ca^2+^) channels, such as NMDA, STIM-I, T-type, TRP, and IP3, on the cytomembrane and the endoplasmic reticulum may induce an increase in cytosolic Ca^2+^ in cancer cells [4–7]. The upregulation of Ca^2+^ signaling in cancer cells contributes to the development of malignant phenotypes, including proliferation, immortalization, angiogenesis, invasion, immune evasion, and drug resistance [8].

Ca^2+^ is a ubiquitous second messenger that serves as a signaling molecule for a variety of cellular processes, such as control of the cell cycle, apoptosis, and cerebellar synapse function, and is simultaneously related to peristalsis, secretion, and immunity [9]. The cytosolic concentration of Ca^2+^ needs to remain constant, and the equivalent amount of imported Ca^2+^ has to be continuously removed from the cytoplasm. Two kinds of transporters are responsible for the elimination of cytoplasmic Ca^2+^. One is the Ca^2+^-ATP pump (PMCA), which is very sensitive to oxidative stress [10]. In terms of quantity, the PMCA has a secondary effect on the overall regulation of neuronal Ca^2+^ [11]. The other transporter is called the Na^+^/Ca^2+^ exchanger (NCX). NCXs operate with a far higher turnover rate than PMCA and are considered to have a stronger ability to discharge cytoplasmic Ca^2+^ in many organizations [12, 13]. NCX proteins mediate the uphill Ca^2+^ outflow in exchange with the downhill Na+ transport, with a ratio of 3 Na^+^:1 Ca^2+^_._ However, under certain altered conditions, such as high intracellular Na^+^ and high positive membrane potential, NCXs may work in the reverse mode and induce Ca^2+^ influx [14].

Three isoforms of NCXs have been found in mammals: NCX1, NCX2, and NCX3. NCX1 is distributed in several tissues and has been extensively studied. The NCX2 and NCX3 genes, however, were mainly found in the central nervous system [15–17]. Therefore, research on NCXs has focused on the heart and brain [18, 19]. To date, some functional studies have explored to the roles of NCXs in the field of cancer in the breast, lung, esophageal, and prostate [20, 21], but little is known about the role of NCXs in CC. The carrier-mediated calcium uptake mechanism exists in the epithelial cells of human colon glands, and NCX was found in the human colon epithelium [22] as a plasma membrane transporter that removes Ca2+ or allows the entry of Ca^2+^ in the cell and maintains cell calcium homeostasis. In vitro rat experiments also revealed that NCXs existed in the apical area of colonic epithelial cells [23]. NCX1 and NCX2 were expressed in nerve cells of the muscle layer of mice. NCX1 can increase colonic peristalsis by increasing the release of acetylcholine in intestinal nerve cells [24]. Upregulation of NCX1 through the response induced by spot stimulation in the vertical smooth muscle showed that transgenic mice had stronger transient colonic relaxation than wild-type mice, and NCX1 was triggered by the NO/sGC/PGG signaling pathway [25]. In the pathological environment of intestinal malignant tumors, the original calcium homeostasis was broken, forming a “new homeostasis” in which the intracellular calcium ion concentration of colorectal gland epithelial tumors increased [26, 27]. However, the role of the expression levels of NCXs in the [Ca^2+^]i increase in promoting the invasion and metastasis of CC cells is still controversial.

It was reported earlier that N-dimethyl-D-erythro-sphingosine (DMS), an inhibitor of protein kinase C and sphingosine kinase, can induce an increase in the intracellular calcium concentration ([Ca^+^]i) in HCT116 colon cancer cells, which suggested that NCX was positively involved in DMS-induced [Ca^2+^]i increase by using NCX inhibitors a priori and NiCl2 to block NCX’s reverse transport of calcium ions into the cell to reduce [Ca^2+^]i [26]. On the one hand, the expression level of NCX was not clear. On the other hand, bepridil (a non-NCX-specific inhibitor) inhibited both receptor-operated calcium channels and voltage-operated calcium channels in vascular smooth muscle, as well as potassium currents and intracellular Ca^2+^/calmodulin complexes [28–30]. NiCl_2_ also has multiple roles, non-specifically regulating calcium channels [31, 32]. A study using next-generation sequencing to reduce the expression levels of 77 transcriptional genes related to cytoplasmic Ca^2+^ transport found that only NCX1 and NCX2 were expressed in NCM460 normal human colon cells and HT29 human colon cancer cells. In tumor cells, there was no difference in the expression of NCX1, while the expression of NCX2 was significantly enhanced [33]. The sodium channels TRPM4 and NCX jointly regulate Ca^2+^-induced mucin secretion in goblet cells [34]. Fourbon Y found that the upregulation of the voltage-gated Ca^2+^ channel (CaV) Ca^2+^ protein α1D can reduce the excretion of Ca^2+^ and increase [Ca^2+^]i by downregulating NCX1/3, leading to the proliferation and migration of the HCT116 colon cancer cell line; moreover, the use of the NCX inhibitor SEA0400 significantly promoted the migration of CRC cells [35]. There are few studies on NCX in CC, and the current studies are basically limited to in vitro studies of cells.

Since the invasion and metastasis of CC are closely related to the level of intracellular Ca^2+^ [36] and NCXs are important proteins involved in the regulation of cytoplasmic Ca^2+^, it is important to observe the expression of NCXs in CC and analyze the relationship between NCXs and CC. For this purpose, we collected 111 clinical cases of stage II–IV colonic tumor tissues and their distal colonic normal tissues and then measured the expression of NCX1, NCX2, and NCX3 by quantitative polymerase chain reaction (qPCR), western blotting (WB), and immunohistochemistry (IHC). The relationships between the expression of NCXs and the prognosis of CC were analyzed by Cox regression analysis and receiver operating characteristic (ROC) curve analysis. To observe the relationship between NCXs and the metastasis of CC, we compared the expression of NCXs in tissues from primary sites, lymph node metastases, and liver metastases.

## Materials and methods

### General information

We collected the clinical data of 111 patients with stage II–IV CC, including 61 patients with lymph node metastasis and 19 patients with liver metastasis, who were admitted to Shengjing Hospital of China Medical University from March 2013 to March 2018. Their pathological specimens were obtained from the pathology department of our hospital. The age range was 43–84 years, and the median age was 62 years. The resected specimens were matched primary colonic malignant tumors and their normal colonic tissue 10 cm away from the tumor. No neoadjuvant therapy was administered. According to the 7th Edition of the American Joint Committee on Cancer (AJCC) staging system [37], 48, 44, and 19 patients were in stage II, III, and IV, respectively. Stage II patients received single-agent chemotherapy based on capecitabine (XELOX) or fluorouracil, stage III patients received the CAPOX or FOLFOX regimen with capecitabine or fluorouracil and oxaliplatin as the main drugs, stage IV patients additionally received targeted therapy such as cetuximab or bevacizumab, and the chemotherapy regimen was changed according to the patient’s condition. Disease-free survival (DFS) was calculated from the operation date to clinical tumor recurrence or metastasis, and overall survival (OS) was calculated from the operation date to the end of the follow-up. According to the National Comprehensive Cancer Network (NCCN; Version 2.2020) guidelines [38], the patients were followed up every 3–6 months in the first 2 years and then every 6–12 months. The end date of the follow-up was March 2020, and the follow-up time was 24–84 months (median 66 months).

Some of the fresh specimens taken from the operation were immediately stored in liquid nitrogen. The others were fixed in formalin. We extracted proteins and RNAs for relevant detection after obtaining a certain number of samples (no more than 6 months) to ensure the freshness of the samples.

### WB analysis

The specimens were ground to a powder after freezing by liquid nitrogen, and the powder was transferred to centrifugal tubes. The proteins were extracted by RIPA lysis buffer with 1 mM PMSF (Beyotime, Shanghai, P.R. China). The protein concentration was measured by the BCA method. The same amount of protein (50 μg) was separated by 8% SDS-PAGE and then transferred to PVDF membranes. The blot membranes were blocked with 5% skim milk for 2 h and then mouse anti-NCX1 monoclonal antibody (1:2000, Abcam, Cambridge, UK), rabbit anti-NCX2 polyclonal antibody (1:2000, Bioss, Beijing, P.R. China) and goat anti-NCX3 polyclonal antibody (1:2000, Santa Cruz Biotechnology, Santa Cruz, CA, USA) were added and shaken at 4 °C overnight. Horseradish peroxidase-labeled secondary antibody was added and incubated for 2 h. The proteins were detected by a biomolecule ChemiDoc^TM^ imaging system (Bio-Rad, CA, USA). The ratio of the gray value of the target protein to GAPDH was used as the relative expression of the target protein. ImageJ software v1.52 (NIH, Bethesda, MD, USA) was used for semi-quantitative analysis.

### qPCR analysis

RNA was purified from the tissue samples by TRIzol Reagent (Thermo Fisher Scientific, Waltham, MA, USA). The nucleic acid concentration was determined using a Nanodrop One spectrophotometer (Thermo, USA) and adjusted to 250 ng/μl. The RNA was then reverse-transcribed into cDNA by the PrimeScript^TM^ RT Reagent Kit (Takara, Shiga, Japan). qPCR was performed using SYBR® Premix Ex Taq™ II (Takara, Ltd.) in an observation system (Roche 480 Diagnostics GmbH, Mannheim, Germany) under the following thermocycling conditions: 95 °C for 35 s, followed by 40 cycles of 95 °C for 5 s and 60 °C for 34 s, 95 °C for 15 s, 60 °C for 34 s, and 95 °C for 15 s. Quantitative gene expression was quantified with the 2^−ΔΔCT^ comparative method, and the final result was analyzed statistically and plotted using GraphPad Prism 6.0 (GraphPad Software Inc., CA, USA). GAPDH was quantified as an internal control. The primer sequences were as follows: NCX1 Forward: 5′-GCC CTG TTA TTG AAT GAG CTT G-3′, Reverse: 5′-TTC CTC TTT GCT GGT CAG TG-3′; NCX2 Forward: 5′-GAA CTT GGC CTT GGT AAT TGG-3′, Reverse: 5′-GTC AGG AAG TGC ATC ACG TAG-3′; NCX3 Forward: 5′-AAG ACT ACG GTG GAC AAA CTG-3′, Reverse: 5′-GAT TCA TCC TCA TCC TCA TCC C-3′; and GAPDH, Forward: 5′-GGA GCG AGA TCC CTC CAA AAT-3′, Reverse: 5′-GGC TGT TGT CAT ACT TCT CAT GG-3′.

### IHC staining

The paraffin specimens (primary tumor, lymph node metastasis, and liver metastasis specimens from the same patient with CC) were cut into 5-μm sections. After deparaffinization and dehydration, antigen repair was performed in 10 mmol/L citrate buffer (pH 6.0) for 10 min in a microwave oven. The sections were incubated with 3% hydrogen peroxide at room temperature (RT) for 40 min to eliminate endogenous peroxidase, and non-specific binding was blocked with 4% goat serum for 40 min. The sections were incubated with mouse anti-NCX1 monoclonal antibody (1:400, Abcam, Cambridge, UK), rabbit anti-NCX2 polyclonal antibody (1: 200, Bioss, Beijing, P.R. China), goat anti-NCX3 polyclonal antibody (1:200, Santa Cruz, CA, USA), rabbit anti-CD3 polyclonal antibody (1:800, Proteintech, Wuhan, P.R. China), mouse anti-CD20 monoclonal antibody (1:800, Proteintech, Wuhan, P.R. China), and mouse anti-CD68 monoclonal antibody (1:400, Proteintech, Wuhan, P.R. China) at 4 °C overnight. Horseradish-labeled goat anti-mouse, goat anti-rabbit, and rabbit anti-goat secondary antibodies (Zsgb-Bio, Beijing, P.R. China) were added for 2 h at RT according to the species of the primary antibodies. DAB working solution (Zsgb-Bio, Beijing, P.R. China) was used to label positive cells. The results were observed and recorded by optical microscopy (DMD108, Leica, Germany). According to the IHC scoring system, each section was scored based on five random fields of magnification (× 400). Image-Pro Plus software 6.0 (Media Cybernetics, Rockville, USA) was used for expression intensity analysis.

### Kaplan-Meier survival curve analysis

Among 111 patients with CC, the correlations between the two groups with high and low expression levels of NCX1, NCX2, and NCX3 and OS were analyzed by Kaplan-Meier survival curves. The same method was used to analyze the correlations between NCXs and DFS.

### Cox analysis

We enrolled 111 patients with colon cancer tumor tissue (tumor) and adjacent normal colon tissue (normal) to detect the expression levels of NCX proteins. Comparing the tumor and normal tissues of each patient, NCX expression levels in tumor tissues greater than those in normal tissues were defined as the NCX high expression group, and NCX expression levels in tumor tissues less than those in normal tissues were defined as the NCX low expression group according to the semi-quantitative results of WB. A total of 111 tumor/normal ratios were obtained, indicating the degree of downregulation or upregulation of NCXs in each patient. The degree of NCX downregulation and patient survival time were used for univariate and multivariate analyses to determine whether NCX downregulation is a prognostic factor affecting CC.

Cox univariate and multivariate regression analyses were used to analyze whether NCXs were independent prognostic factors of CC, and other variables, including sex, age, TNM stage, tumor differentiation degree, tumor size, CEA, and ratio of NCX (NCX1, NCX2, and NCX3) expression levels in tumor tissues to normal tissues (tumor/normal) were also analyzed.

### ROC curve

The expression levels of NCXs in 111 stage II-IV CC patients were analyzed by WB. ROC curves were calculated for the NCX (NCX1, NCX, and NCX3) ratios of tumors to normal tissues according to the previous Cox analysis description to define the NCX cutoff values related to OS. Kaplan-Meier curve analysis, Cox regression analysis, and ROC curve analysis were performed using SPSS statistics 17.0 (SPSS Corporation, Chicago, USA).

### Statistical analysis

The data are presented as the mean ± standard deviation. The difference in the relationship between NCX expression and the clinicopathological features of patients was evaluated using the Mann-Whitney U test. The log-rank test was used to test the differences in Kaplan-Meier survival curves. Cox regression analysis was used to determine the independent prognostic factors of CC. ROC curves were used to quantitatively analyze the relationship between NCXs and OS. To test single variables between two groups, a paired t test was performed. To test single variables between multiple groups, a one-way ANOVA was performed. A *p* value < 0.05 was considered to be significant and is presented as *p < 0.05, **p < 0.01, or ***p < 0.001.

## Results

### The downregulated expression of NCXs in CC

**Fig. 1:**
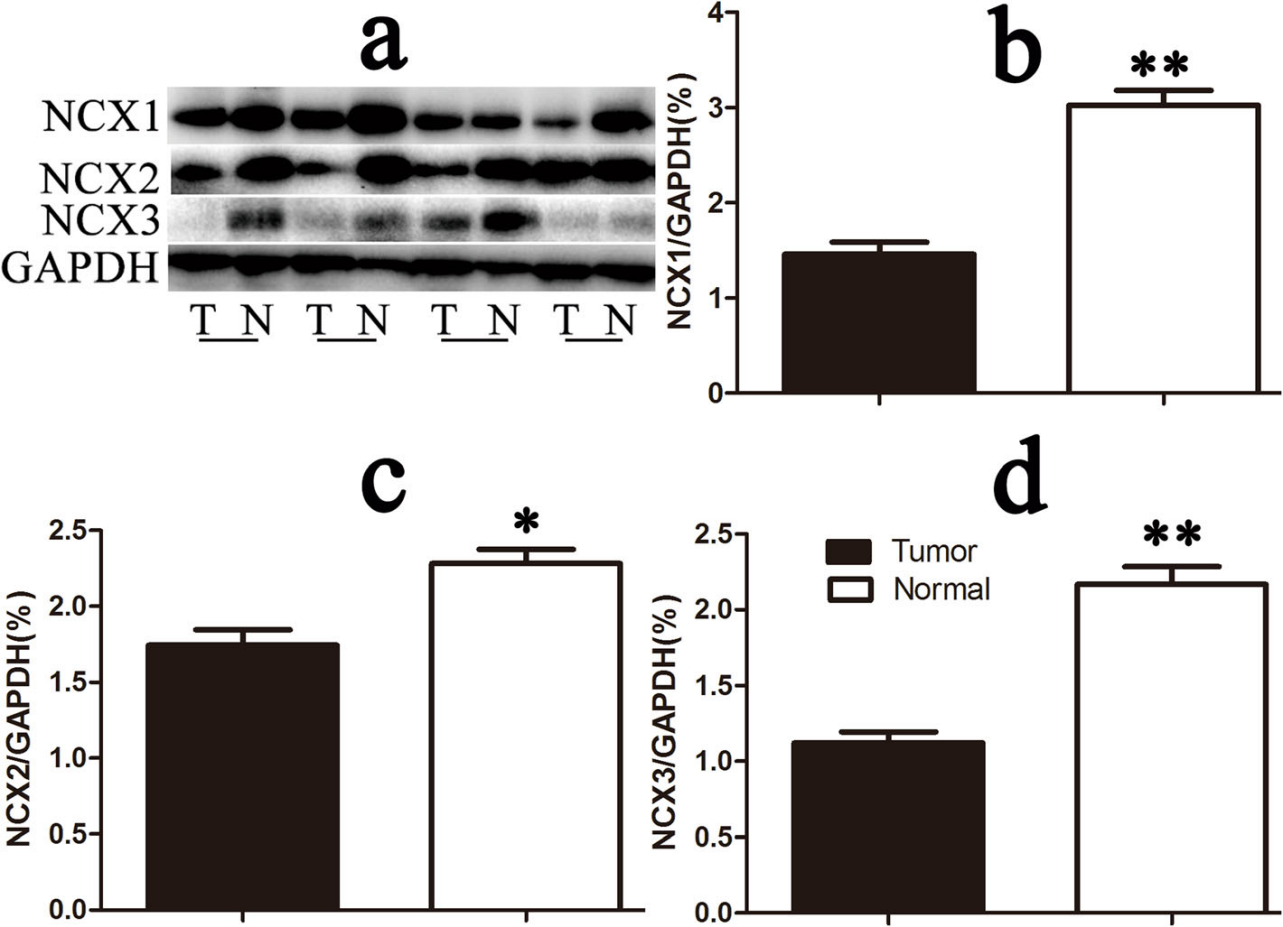
Downregulation of NCX protein expression in colon cancer (CC). **a** Western blotting was used to compare the expression of the three isoforms of NCXs in tumor tissues with that in the normal colon tissue of 111 CC patients. The intensity of each band was analyzed. The NCX1 (**b**), NCX2 (**c**), and NCX3 (**c**) protein levels in tumors, in general, were significantly lower than those in normal tissue, particularly those of NCX1 and NCX3. The ratio of NCX to GAPDH is shown by a histogram. (T: colonic malignant tumor; N: normal colonic tissue; paired t test; *p < 0.05 and **p < 0.01 compared with T)

**Fig. 2:**
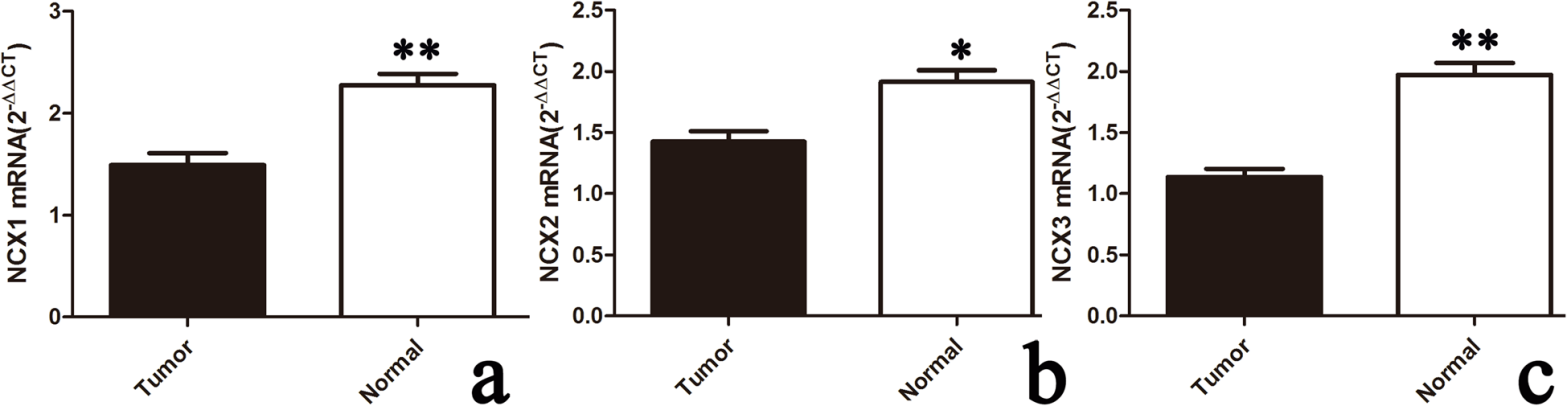
NCX mRNA expression was decreased in CC. **a**–**c** In 111 patients, the overall expression of NCX mRNA in most tumor samples was lower than that in normal tissues by qPCR. Compared with the three isoforms, the mRNA of NCX1 (**a**) and NCX3 (**c**) in tumors decreased more remarkably than that of NCX2 (**b**). Histograms representing the relative expression of NCX compared to GAPDH (paired t test; *p < 0.05, **p < 0.01)

Our WB results showed that the expression of NCXs was generally lower in tumor tissues (tumor) than in normal tissues (normal) (*p* < 0.05) (Fig. 1). The expression of NCX was defined as the low expression (low) group when the ratio of tumor to normal was less than 1. It was defined as the high expression (high) group when the ratio was more than 1. By comparison, we found that the expression levels of NCX1, NCX2, and NCX3 in tumors were low in 75, 81, and 84 patients, which accounted for 67.6%, 73%, and 75.5% of the 111 total patients, respectively. The mRNA expression of the three NCX isoforms was consistent with the WB results (Fig. 2).

### The distribution of NCXs in CC and the normal colon

**Fig. 3:**
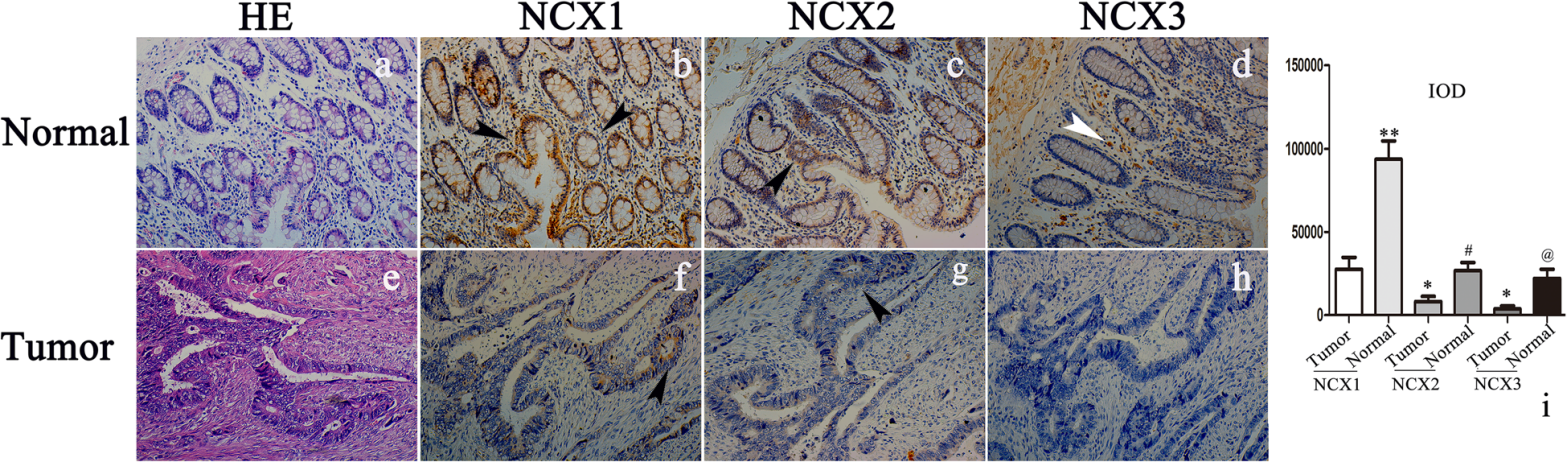
Expression of NCX1, NCX2, and NCX3 in normal and tumor tissues of CC by HE and IHC stainings (×200). **a** HE staining for normal colonic mucosa. The structure of normal colonic mucosa. The glands were arranged in order. The nuclei of epithelial cells and stromal cells were clear. **b** In the normal colon, NCX1 was highly expressed in the cell membrane of normal colonic glandular epithelial cells and stromal cells in the mucosa, and the closer to the intestinal cavity, the stronger the staining was. **c** NCX2 was moderately expressed in stromal cells and glandular epithelium. **d** The expression of NCX3 mainly in stromal cells and submucosa. **e** HE staining for colonic malignant glands. The glands showed disorganized malignant growth. The nucleus was large and hyperchromatic with vacuoles. **f** NCX1 was moderately expressed in malignant glands. **g** The positive expression of NCX2 was noticeably decreased compared to that in normal tissues. **h** The staining intensity of the NCX3 protein in tumor tissue was very low by IHC. **i** The integral optical density (IOD) is represented by a histogram. (tumor: colonic malignant tumor; normal: normal colonic tissue; black arrows refer to glands, white arrows refer to stromal cells; one-way ANOVA; *p < 0.05 and **p < 0.01, compared with NCX1-tumor; ^#^*p* < 0.05, compared with NCX2-tumor; ^@^*p* < 0.05, compared with NCX3-tumor)

The IHC results showed that NCXs were highly expressed in the cell membrane of normal colonic glandular epithelial cells and stromal cells in mucosa, and stronger NCX expression was seen closer to the intestinal cavity. The distributions of NCX2 and NCX3 were similar to that of NCX1, but the degrees were significantly lower than that of NCX1 (Fig. 3a–d).

In the malignant glands of CC, the cells lost polarity, were arranged in a disorderly manner, and exhibited nuclear hypertrophy with vacuolar degeneration, the brush edge of microvilli disappeared, and inflammatory cell infiltration in the matrix was common (Fig. 3e). The expression of NCXs in malignant glands was decreased significantly (Fig. 3f–h). It was difficult to identify the expression of NCX3 in the tumors of some specimens.

### The status of NCXs in immune cells

**Fig. 4:**
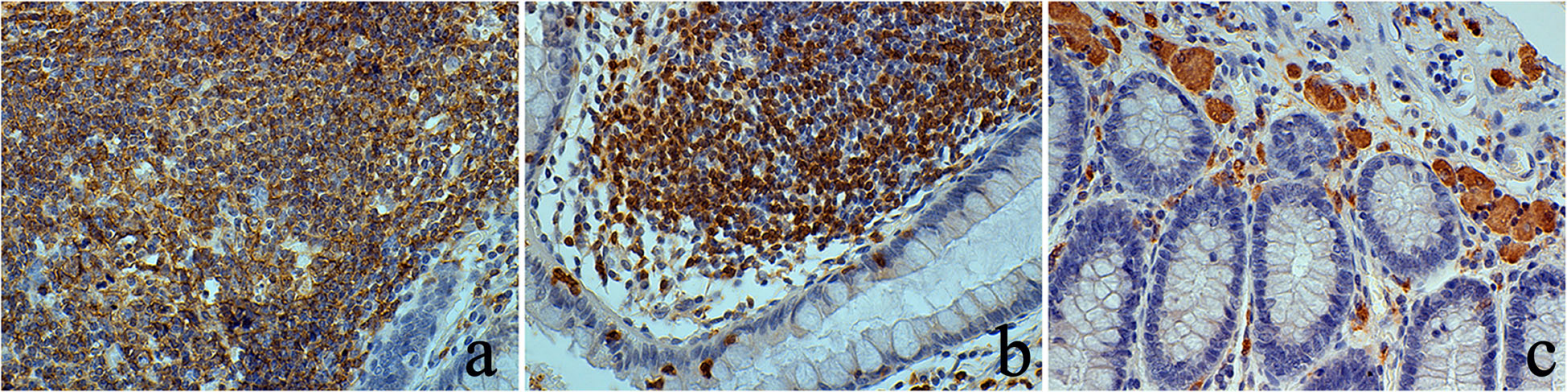
Expression of CD20, CD3, and CD68 in immune cells of the normal colon (×400). **a** The expression of CD20 in B cells of normal colonic lymphoid follicles. **b** The expression of CD3 in T cells of normal colonic lymphoid follicles. **c** CD68 was strongly expressed in macrophages of colonic submucosa

IHC staining of CD3, CD20, and CD68 was used to detect T cells, B cells, and macrophages, respectively. As shown in Fig. 4, CD3 and CD20 were strongly expressed in normal lymphoid follicles, indicating that lymphoid follicles were mainly composed of T cells and B cells. However, CD68 was mainly distributed in submucosal cells with a large volume, indicating that these cells were macrophages. Together, T cells, B cells, and macrophages maintain the immune function of the colon.

**Fig. 5:**
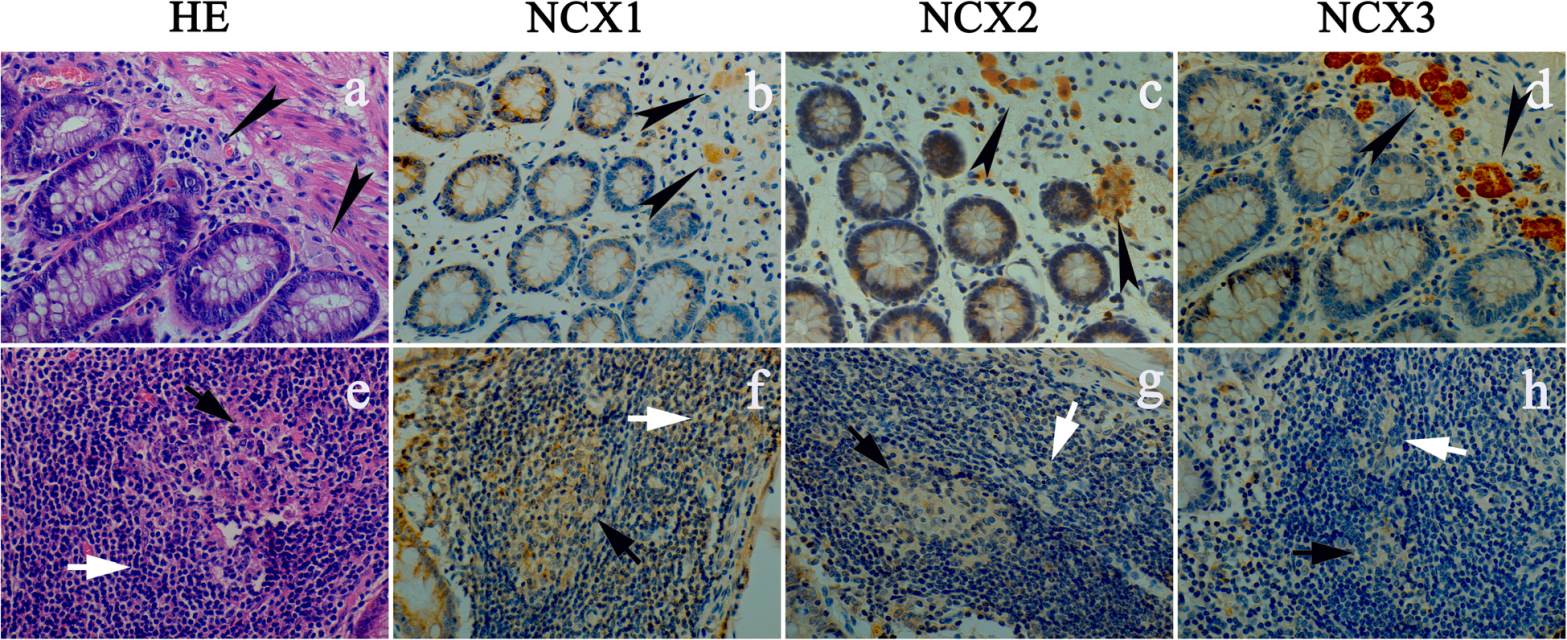
Expression of NCX1, NCX2, and NCX3 in different immune cells of the normal colon (×400). **a** Some macrophages (black dovetail arrows) were found in the submucosa of the normal colon. **b** The protein expression strength of NCX1 in macrophages was medium but weaker than that of NCX2 (**c**). **d** Intense expression of NCX3 in macrophages. **e** In lymphoid follicles of the normal colon, B cells and T cells are indicated by black arrows and white arrows, respectively. **f** The expression of NCX1 was intense in lymphocytes (B cells and T cells). **g** NCX2 was mainly moderately expressed in lymphocytes. **h** NCX3 was weakly expressed in lymphocytes

**Fig. 6:**
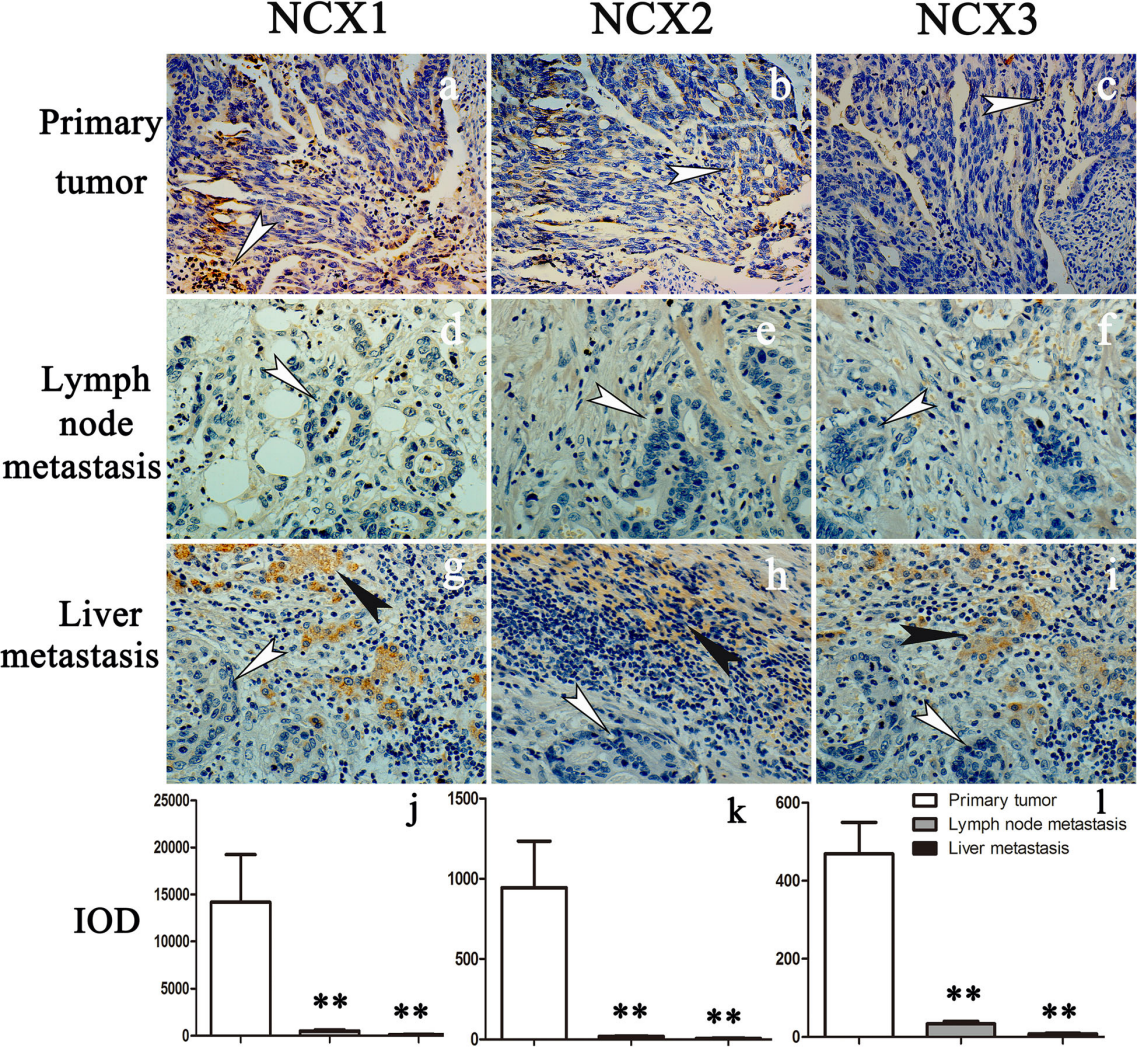
Expression of NCX1, NCX2, and NCX3 in primary and metastatic sites. (×400). **a**–**c** In the primary tumor site, heteromorphism of the glands was very obvious, and the degree of differentiation was moderate, as determined. The expression strengths of NCX1 (**a**), NCX2 (**b**), and NCX3 (**c**) in colonic malignant glands decreased more obviously. **d**–**f** The expression intensity of NCXs at lymph node metastasis sites decreased more significantly than that at primary sites. **d** The expression of NCX1 was weak in malignant glands compared with lymph node metastasis sites. There were nearly no positive NCX2 (**e**) and NCX3 (**f**) cells at the lymph node metastasis sites. **g**–**i** The expression of NCXs in normal hepatocytes (black arrows) at liver metastasis sites was obvious, and the strength of NCX1 staining (**g**; white arrow) was higher than that of NCX2 (**h**; white arrow) and NCX3 (**i**; white arrow). NCXs were almost not expressed in malignant glands (**g**, **h**, **i**; white arrows). The IOD is represented by a histogram (**j**–**l**)

As shown in Fig. 5, NCX3 was highly and specifically expressed in macrophages between the glandular epithelium and the muscularis mucosa of the normal mucosal layer in some specimens. The expression of NCX3 was the strongest compared with that of the other isoforms, whereas the expression of NCX1 was the weakest. In lymphoid follicles (Fig. 6), NCXs were expressed in the cell membrane of central B cells and peripheral T cells, while the expression of NCX1, NCX2, and NCX3 decreased. These data suggested that NCXs may be involved in normal intestinal immune function.

### Low expression of NCXs was associated with the invasion and metastasis of CC

We compared the expression of NCXs in the primary sites to that in the metastatic sites of the same patients. In the primary site of the tumor, the heteromorphism of glands was very obvious. The expression of NCX1 and NCX2 in malignant glandular epithelial cells was weak. NCX3 was found only in a few stromal cells. The weak NCX1 signal and almost absent NCX2 and NCX3 signals were observed in the malignant glands of the metastatic lymph nodes. In the samples of liver metastases from CC, the expression of the three isoforms in residual liver cells was different, and the expression of NCX1 was stronger than that of the others. However, it was difficult to identify the signals of NCXs in metastatic malignant glands. The results of the expression analyses of NCXs and clinicopathological characteristics also showed that NCXs were negatively correlated with invasion and metastasis indicators such as lymph node and liver metastases of CC (Table 1).

**Table 1:** Correlation between NCXs and the clinicopathological features of colon cancer

Clinicopathological feature	NCX1 expression				NCX2 expression				NCX3 expression			
	Case	T/N < 1^**a**^	T/N ≥ 1^**b**^	*P* value	Case	T/N<1^**a**^	T/N≥1^**b**^	*P* value	Case	T/N < 1^**a**^	T/N ≥ 1^**b**^	*P* value
	*n* = 111	*n* = 74	*n* = 37		*n* = 111	*n* = 81	*n* = 30		*n* = 111	*n* = 84	*n* = 27	
Sex												
Male	68	50	18	0.054	68	47	21	0.25	68	53	15	0.484
Female	43	24	19		43	34	9		43	31	12	
Age, years												
< 60	72	45	27	0.206	72	49	23	0.113	72	57	15	0.244
≥ 60	39	29	10		39	32	7		39	27	12	
T stage^c^												
T2	8	5	3	0.948	8	6	2	0.738	8	6	2	0.571
T3	65	44	21		65	49	16		65	47	18	
T4	38	25	13		38	26	12		38	31	7	
N stage^c^												
N0	50	30	20	0.177	50	32	18	0.054	50	31	19	0.002**
N1	61	44	17		61	49	12		61	53	8	
M stage^c^												
M0	92	56	36	0.004*	92	64	28	0.075	92	66	26	0.033*
M1	19	18	1		19	17	2		19	18	1	
Differentiation												
High	13	9	4	0.068	13	10	3	0.574	13	8	5	0.448
Medium	72	43	29		72	51	21		72	56	16	
Low	26	22	4		26	20	6		26	20	6	
Tumor size												
≥ 5 cm	52	37	15	0.283	52	37	15	0.685	52	39	13	0.876
5 cm	59	35	22		59	44	15		59	45	14	
CEA												
Positive	58	40	18	0.591	58	44	14	0.473	58	44	14	0.962
Negative	53	34	19		53	37	16		53	40	13	

^a^The expression level of NCX in tumor tissues is less than 1 compared with that in normal tissues

^b^The expression level of NCX in tumor tissues is greater than 1 compared with that in normal tissues

^c^TNM stage; *N1* lymph node metastasis, *M1* distant metastasis; *p < 0.05, **p < 0.01

### The downregulation of NCXs was associated with the prognosis of CC

According to the follow-up data of the 111 patients, 92 patients (82.8%) did not experience metastasis, 19 patients (17.1%) had distant metastasis before the operation, 56 patients (50.5%) survived disease-free at the end of the follow-up, 55 patients (49.5%) died, and 34 patients (30.6%) had new local recurrence and distant metastasis postoperatively. The mean OS of the 111 patients was 55.54 ± 2.69 months (95% CI 50.27 ~ 60.80), and the mean DFS was 68.23 ± 3.47 months (95% CI 40.03 ~ 53.62). The mean OS of the 55 patients who died was 33.73 ± 2.56 months (95% CI 28.70 ~ 38.75), and the mean time to recurrence and metastasis was 12.87 ± 2.35 months (95% CI 8.27 ~ 17.48). Among them, 34 patients had new recurrence and metastasis after surgery, with a mean OS of 38.59 ± 3.38 months (95% CI 31.96 ~ 45.22), and a mean time to recurrence and metastasis of 20.82 ± 3.10 months (95% CI 14.75 ~ 26.90).

**Fig. 7:**
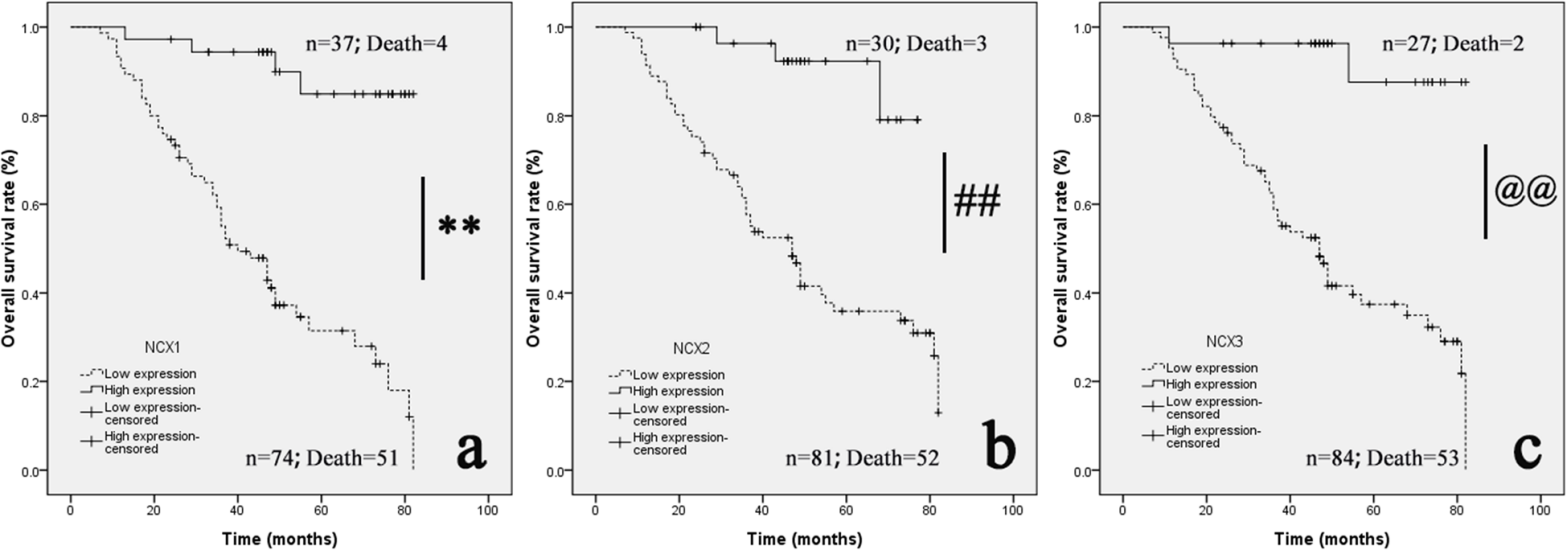
Decreased NCX expression was negatively correlated with the overall survival (OS) time. **a** Kaplan-Meier curves of the OS of 111 CC patients are displayed according to the NCX1 low and NCX1 high expression groups. **b** Kaplan-Meier survival analysis curves are also displayed for different NCX2 expression groups. **c** Kaplan-Meier survival analysis curves are also displayed for different NCX3 expression groups. The log-rank test indicated a significant difference between the survival curves (**p < 0.01; ##*p* < 0.01; @@*p* < 0.01)

In the survival analysis of the mean OS in 111 patients with NCXs, the OS for NCX1 in the low group was 45.75 ± 3.09 months (95% CI 39.71 ~ 51.80), and that of the high group was 75.73 ± 2.96 months (95% CI 69.95 ~ 81.53); the difference between the two groups was statistically significant (*P* < 0.01) (Fig. 7a). The OS for NCX2 in the low group was 48.74 ± 3.10 months (95% CI 42.67 ~ 54.81), and that in the high group was 72.67 ± 2.36 months (95% CI 68.04 ~ 77.30); the difference between the two groups was statistically significant (*P* < 0.01) (Fig. 7b). The OS for NCX3 in the low group was 49.24 ± 3.0 months (95% CI 43.35 ~ 55.13), and that in the high group was 76.92 ± 3.41 months (95% CI 70.23 ~ 83.61); the difference between the two groups was statistically significant (*P* < 0.01)(Fig. 7c). The experimental results showed that in terms of NCXs, patients in the low group had a shorter OS.

**Fig. 8:**
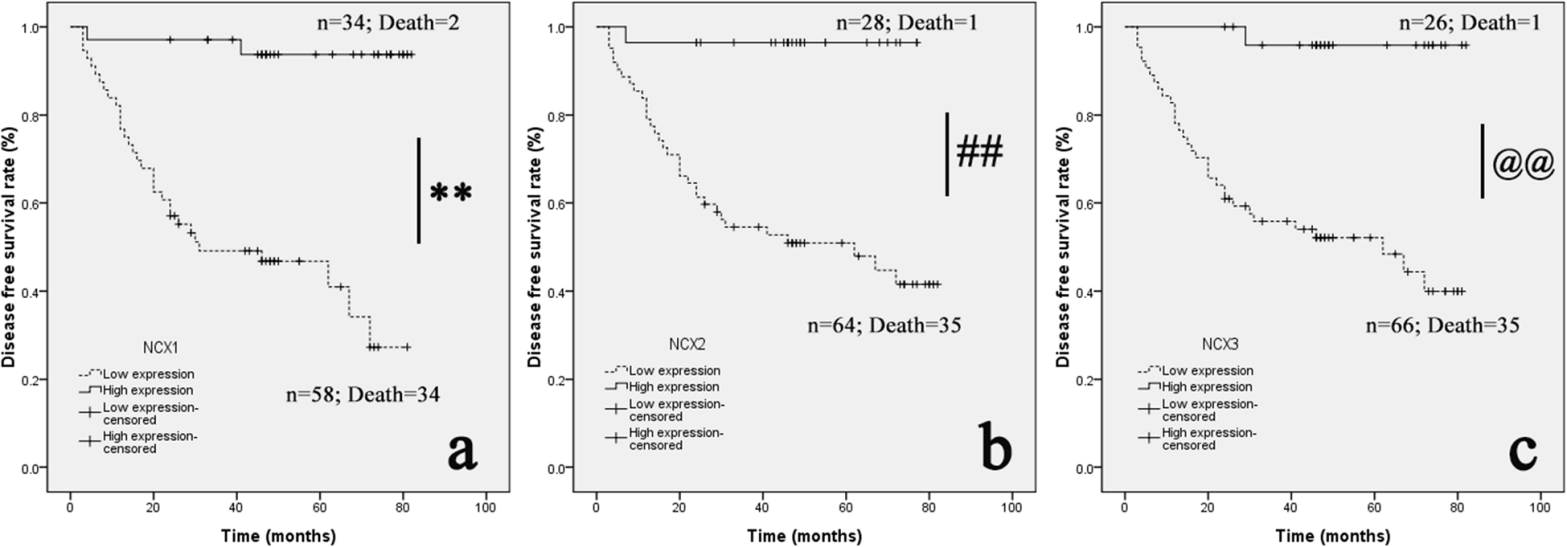
Decreased expression of NCXs was negatively correlated with the disease-free survival (DFS) time. **a** Kaplan-Meier curves of the DFS of 92 preoperative non-metastatic CC patients are displayed according to the NCX1 low and NCX1 high expression groups. **b** Kaplan-Meier survival analysis curves are also displayed for different NCX2 expression groups. **c** Kaplan-Meier survival analysis curves are also displayed for different NCX3 expression groups. The log-rank test indicated a significant difference between the survival curves (**p < 0.01; ##*p* < 0.01; @@*p* < 0.01)

In the survival analysis of DFS in 92 patients with NCXs, the OS for NCX1 in the low group was 44.00 ± 4.29 months (95% CI 35.59 ~ 52.41), and that in the high group was 78.33 ± 2.60 months (95% CI 73.24 ~ 83.42); the difference between the two groups was statistically significant (*P* < 0.01) (Fig. 8a). The value of the NCX2 in the low group was 48.71 ± 4.17 months (95% CI 40.54 ~ 56.88), and that in the high group was 74.50 ± 2.46 months (95% CI 69.69 ~ 79.31); the difference between the two groups was statistically significant (*P* < 0.01) (Fig. 8b). The OS for NCX3 in the low group was 48.42 ± 4.08 months (95% CI 40.42 ~ 56.42), and that in the high group was 79.79 ± 2.16 months (95% CI 75.55 ~ 84.03); the difference between the two groups was statistically significant (*P* < 0.01) (Fig. 8c). The experimental results showed that in terms of NCXs, patients in the low group had a shorter DFS.

### NCXs can be used as independent prognostic factors of CC

Cox univariate and multivariate regression analyses were used to analyze whether NCXs were independent prognostic factors of CC. The univariate analysis showed that sex, age, and tumor size had no significant effect on OS (P > 0.05); on the other hand, differentiation degree (*P* < 0.05), CEA (*P* < 0.05), T stage (*P* < 0.05), lymph node metastasis (*P* < 0.01), distant metastasis (*P* < 0.01), and NCX expression (*P* < 0.01) affected the OS of CC (Table 2). The multivariate Cox regression analysis showed that T stage (*P* < 0.05), lymph node metastasis (*P* < 0.05), distant metastasis (*P* < 0.01), NCX3 expression (*P* < 0.05), and NCX1 and NCX2 expression (*P* < 0.01) were independent prognostic factors of CC (Table 2). Therefore, decreased NCX expression may indicate poor prognosis, invasion, and metastasis in CC.

**Table 2:** Cox univariate and multivariate survival analyses to analyze the independent prognostic factors for colon cancer

Characteristics	HR	Univariate CI (95%)	*P*	HR	Multivariate CI (95%)	*P*
Sex						
(Male/female)	1.075	0.602, 0.337	0.086	0.774	0.419, 1.427	0.411
Age, years						
(≥ 60/< 60)	0.883	0.022, 1	0.883	0.87	0.473, 1.599	0.653
TNM stage						
T stage	2.584	1.601, 0.992	< 0.05*	1.779	1.019, 3.106	< 0.05*
N stage	10.801	5.501, 2.801	< 0.01**	2.285	1.057, 4.938	< 0.05*
M stage	13.214	7.309, 4.043	< 0.01**	2.984	1.594, 5.587	< 0.01**
Differentiation degree						
(high/medium/low)	3.141	1.924, 1.178	< 0.05*	0.801	0.442, 1.451	0.464
Tumor size						
(≥ 5 cm/< 5 cm)	1.295	0.762, 0.448	0.315	1.491	0.755, 2.944	0.25
CEA						
(Positive/negative)	0.996	0.578, 0.336	< 0.05*	0.778	0.421, 1.44	0.425
NCX1						
(Tumor/normal)	0.277	0.099, 0.036	< 0.01**	0.145	0.049, 0.43	< 0.01**
NCX2						
(Tumor/normal)	0.422	0.132, 0.041	< 0.01**	0.187	0.056, 0.628	< 0.01**
NCX3						
(Tumor/normal)	0.357	0.087, 0.021	< 0.01**	0.215	0.05, 0.936	< 0.05*

Tumor/normal: the ratio of the NCX expression level in tumor tissues to that in normal tissues; *p < 0.05; **p < 0. 01

**Fig. 9:**
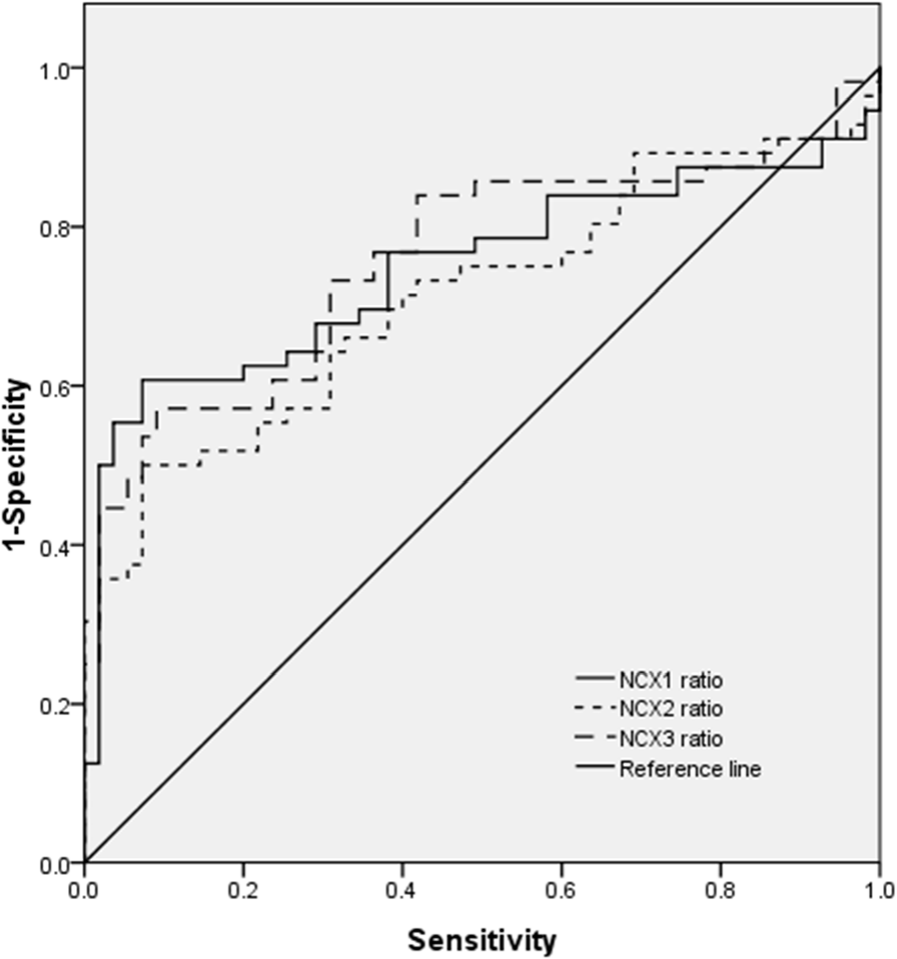
NCXs can predict the prognosis of CC. According to the grayscale value of NCX expression in tumor tissue (tumor) and matched normal tissue (normal) calculated by WB bands, the ratios (tumor/normal) of NCX were obtained. Three group ratios of NCX1, NCX2, and NCX3 (*n* = 111; NCXs were identified by different curves) were used to draw ROC curves. The ROC curves used the NCX (NCX1, NCX, and NCX3) ratios to define the NCX cutoff values related to OS. The ROC curves of NCX1, NCX2, and NCX3 were used to predict the OS of 111 CC patients. The abscissa represents sensitivity, and the ordinate represents specificity. Numerical analysis of the area under the ROC curve showed significant differences

We used ROC curves to assess the predictive ability of the downregulation ratio (NCXs-T/NCXs-N) for OS in patients with CC (Fig. 9). The area under the ROC curve of NCX1 was 74.7% (95% CI 0.65–0.844), the optimal critical value was 0.96, the specificity was 60.7%, and the sensitivity was 92.7%. The area under the ROC curve of NCX2 was 71.2% (95% CI 0.614–0.809), the optimal critical value was 0.97, the specificity was 50.0%, and the sensitivity was 92.7%. The area under the ROC curve of NCX3 was 75.9% (95% CI 0.667–0.852), the optimal critical value was 0.92, the specificity was 57.1%, and the sensitivity was 99.0%. The expression of NCX3 in colon tumors was the most significantly decreased compared with that in normal colon tissues (*P* < 0.05). Although the three NCXs can predict OS, NCX1 had the strongest specificity, and NCX3 had the highest sensitivity. NCXs can predict the prognosis of CC, and their downregulation was associated with relatively short OS times.

## Discussion

NCXs, which are the homeostasis control channel of calcium ions inside and outside the cell, have been studied in myocardial ischemia-reperfusion injury and nerve function [39–41], and information on them has expanded in tumors in recent years and many other fields [42–44]. NCXs participate in the normal physiological functions of the colon, in which calcium ions are involved in peristalsis, the secretion of intestinal liquid and factors, and nerve regulation [24, 45, 46]. In normal cells, NCXs briefly resist calcium oscillations and can quickly expel intracellular calcium ions to perform their functions, avoid calcium overload, and maintain calcium homeostasis [40, 47]. There are very few reports on the expression and roles of NCX2 and NCX3 in the colon; however, our results showed that the NCX2 and NCX3 proteins were detected in the normal colon. IHC staining indicated that the distribution and expression of the three isoforms were different. NCX1 was highly expressed in the cell membrane of normal colonic glandular epithelial cells and stromal cells in the mucosa. Some studies have confirmed that NCX1 participates in the physiological regulation mechanism of intestinal calcium absorption, bicarbonate secretion, smooth muscle movement, and so on [48]. The expression of NCX2 was similar to that of NCX1, indicating that its function may resemble that of NCX1. Although the expression of NCX3 was lower than that of NCX1 and NCX2 in mucosal glands, it was higher than that in CD68-positive macrophages in the stroma, which indicated that NCX3 may play a defensive role in the colon. Macrophages are an important part of the innate immune system and have a wide range of functions, including phagocytosis, antigen presentation, the secretion of growth factors and cytokines, and so on [49]. In addition, different expression levels of NCXs were also found in the lymphoid follicles of CD3-labeled T cells and CD20-labeled B cells. In contrast, CD staining of positively labeled immune cells almost disappeared in malignant colon tumors. It has been reported that lymphoid follicles have immune-mediated anti-tumor effects [50], and the expression of NCXs suggests that they are likely to be involved in immune function and anti-cancer effects in the intestinal tract.

Our results also showed that in more than two-thirds of cases, the protein and mRNA levels of NCXs were remarkably downregulated in tumors compared with the corresponding normal colon. Through morphological observation and the analysis of clinicopathological relationships (Table 1), we found that the expression of NCXs in the glands of malignant tumors was decreased, and the decline in NCXs was even more pronounced, especially in metastatic lymph nodes and liver metastases, which illustrated that the downregulation of NCXs may be positively related to the malignant biological behavior of CC. To uncover the relationship between the expression of NCXs in tumors and prognosis, 111 patients with stage II–IV CC were divided into low and high groups according to the expression ratios of NCXs in tumor tissues to normal tissues. The statistical data showed that the low NCX1, NCX2, and NCX3 groups had lower survival rates than the high NCX1, NCX2, and NCX3 groups. It was further confirmed that the low expression of NCXs affected the prognosis of CC. The downregulation ratio of NCXs in cancer tissue compared with normal control tissue was an important factor; when it was lower than this critical value, the overall survival time of the patient was shortened. Furthermore, the receiver operating curve analysis results revealed that tumor/normal NCX expression ratios (NCX1: 0.96; NCX2: 0.97; NCX3: 0.92) can be used as independent prognostic indicators of OS.

Research on NCXs has focused on the areas of the heart and brain for a long time; in recent years, articles on sporadic function have linked cancer with NCXs. For example, NCX1 was found to be highly expressed in esophageal cancer and considered to be involved in the pathogenesis of esophageal cancer [51]. The overexpression of NCX3 in ovarian carcinoma cells more easily led to therapy resistance [12]. The common feature of these studies was that the upregulation of NCXs promoted the proliferation of cancer cells. In contrast, our results showed that the downregulation of NCX expression was an important factor affecting the prognosis of CC. The contradictory results may be due to different tissue specificities. Ca^2+^ protein alpha 1D is expressed in a variety of malignant tumors and promotes tumor progression [52]. Inhibiting NCX1/3 and promoting the release of calcium from the endoplasmic reticulum can increase [Ca^2+^]i, upregulate Ca^2+^ protein alpha 1D, and promote the migration of HCT116 colon cancer cells [35]. The physiological function of NCXs is the excretion of Ca^2+^ from the cytoplasm, so the downregulation of NCXs can certainly affect the capacity of Ca^2+^ excretion. Although PMCA in cancer cells can pump out Ca^2+^ from the cytoplasm, its ability was 50–100 times weaker than that of NCXs [40]. Therefore, the downregulation of NCXs may prevent cytoplasmic Ca^2+^ from being released effectively, which results in a high level of Ca^2+^ in CC cells. In the pathological environment of intestinal malignant tumors, the original calcium homeostasis is broken, forming a “new homeostasis” in which the [Ca^2+^]i of colorectal glandular epithelial tumors is increased, and the increase in [Ca^2+^]i activates calmodulin kinase II (CaMKII) and hypoxia-inducible factor-1a (HIF1a), which are involved in tumor progression [26, 27]. The upregulation of Ca^2+^/calmodulin-dependent protein kinase II and HIF1a in CC cells promoting tumor cell invasion and metastasis is related to aerobic glycolysis, and the activation of downstream factors MMPs and EMT also participates in tumor cell migration [53, 54]. Some scholars have found that the mitochondrial Na^+^/Ca^2+^/Li^+^ exchanger (NCLX) is significantly downregulated in patients with colorectal cancer. The reduction in NCLX led to transcriptional changes, and the expression of genes that regulate EMT and cancer stemness increased. Mesenchymal phenotype expression promoted colorectal cancer metastasis and drug resistance. In addition, the decrease or loss of NCLX expression can cause mtCa^2+^ overload, which leads to mitochondrial depolarization, increases mtROS production, initiates the mitochondrial Ca^2+^/ROS signaling axis to drive HIF1a activation and HIF1a-dependent glycolysis, promoting the migration, invasion, and metastasis of colorectal cancer cells [54]. The disappearance of NCX in CC tumor cells indicated that NCX may be a tumor suppressor. Our experimental results supported that NCX and its isomer NCLX had similar inhibitory effects on CC. The reduction in tumor suppressor factors exacerbated the vicious cycle of calcium overload. Calcium overload can activate many channels and factors related to calcium ions. Studies have shown that they can promote the proliferation, invasion, metastasis, and anti-apoptosis of tumor cells in CC [36, 55].

We preliminarily verified that NCX was a tumor suppressor in human colon tumor tissues. Certainly, the current research on the roles of NCXs in CC is still in an early stage, and there is no clarity on the mechanism of NCX regulation. As a next step, we will further verify the tumor suppressor mechanism of NCXs in a variety of in vitro cell lines. However, the three isoforms of NCXs can separately be used as independent prognostic factors of CC as long non-coding RNAs, autophagy-related genes, immune genes, and so on [56–58], which illustrates that they have prospects in the field of advanced CC. With NCX as the target, the calcium ion pathway has good application prospects in the research of new anti-tumor drugs.

## Conclusion

The expression of NCXs was decreased in CC tissues compared to adjacent normal tissues. The degree of downregulation of NCXs was positively correlated with the prognosis of CC. NCX1, NCX2, and NCX3 can be used as independent prognostic indicators of CC.

## Data Availability

Please contact author with requests for data.

## Abbreviations

CC: Colon cancer
NCX: Na+/Ca2+ exchanger
PMCA: Ca2+-ATP pump
ROC: Receiver operating characteristic
AJCC: American Joint Committee on Cancer
DFS: Disease-free survival
OS: Overall survival
NCCN: National Comprehensive Cancer Network
qPCR: Quantitative polymerase chain reaction
WB: Western blotting
IHC: Immunohistochemistry
CaV: Voltage-gated Ca2+ channel
[Ca+]i: Intracellular calcium concentration
CaMKII: Calmodulin kinase II
HIF1a: Hypoxia-inducible factor-1a
